# Not Just a ‘Breath of Death’: Indirect Consequences of Working in a COVID-19 Unit

**DOI:** 10.3390/ijerph182010802

**Published:** 2021-10-14

**Authors:** Anasuya Guha, Petr Schalek, Martin Chovanec, Pavel Kraml, Jakub Bala, Jan Plzak

**Affiliations:** 1 Department of Otorhinolaryngology, General University Hospital in Prague, 128 08 Prague, Czech Republic; 2 Department of Otorhinolaryngology, University Hospital Kralovske Vinohrady, 3rd Faculty of Medicine, Charles University, 100 34 Prague, Czech Republic; petr.schalek@fnkv.cz (P.S.); martin.chovanec@fnkv.cz (M.C.); 3 Department of Internal Medicine, University Hospital Kralovske Vinohrady, 3rd Faculty of Medicine, Charles University, 100 34 Prague, Czech Republic; pavel.kraml@fnkv.cz; 4 Department of Anesthesiology and Resuscitation, University Hospital Bulovka, 180 81 Prague, Czech Republic; jakub.bala@bulovka.cz; 5 Department of Otorhinolaryngology, Head and Neck Surgery, University Hospital Motol, 1st Faculty of Medicine, Charles University, 150 06 Prague, Czech Republic; jan.plzak@fnmotol.cz

**Keywords:** COVID-19, COVID-19 unit, frontiers, psychosomatic problems, mental fatigue

## Abstract

We carried out a survey in the Czech Republic between January and February 2021 to evaluate the impact of COVID-19 on doctors working in the COVID-19 unit. A rise in 250,000 cases were seen in the Czech Republic during the time of the survey. The indirect impact of the disease on doctors working in COVID-19 units and strategies to control the situation in the Czech Republic were evaluated here. About 35% doctors were concerned with health issues, 40% had tested positive for SARS-CoV-2 antigen, 51% reported lack of support for those who had contracted COVID-19 and 163 agreed that medical, psychological counselling and financial services should be provided. Most doctors experienced moderate and severe degrees of psychological impact. Doctors with the least working experience and those with at least 21 to 35 years were most affected. Mental fatigue was the most common reported psychosomatic effect. The effects were higher in doctors who were more concerned about working in COVID-19 units. Around 87% agreed that the best strategy in controlling the situation in the Czech Republic would be ‘preventive measures in combination with vaccination’. History shows us that pandemics can occur in multiple waves. Subsequent waves, inadequate support as well as unparalleled workload can lead to a serious rise in psychological disorders amongst HCWs worldwide.

## 1. Introduction

The airborne viral infection COVID-19 has affected more than 181 million people and caused over 3.9 million deaths across 220 countries and territories since it was first declared as a pandemic by the WHO in 2019 [1]. Three principal ways of being exposed to the virus are inhalation of very small fine respiratory droplets and aerosol particles, deposition of virus during exhalation onto exposed mucous membranes (mouth, nose or eye) and touching mucous membranes with hands contaminated with the virus or touching inanimate surfaces infected with the virus [2]. However, the modes of transmission are varied and therefore any animate or inanimate surface is a potential vector for infection. In effect, nosocomial spread has also increased, leading to temporary closure of entire outpatient services, emergency services and in some cases even large university hospitals (800-bed capacity) [3], thus seriously reducing the already deficient medical resources. Therefore, even with high precautionary measures such as using masks or respirators, lockdown, quarantine, disinfection techniques and so on, disease spread has been unavoidable thus incrementally pressurizing healthcare systems worldwide. Many countries secured funds to help healthcare systems [4,5], but the burden of work on healthcare workers (HCWs) has not been eased. In addition, the multiple waves of COVID-19 and the ongoing development of newer variants of the pathogen make the disease even more difficult to manage and control effectively. Amongst other factors, there is also little time for frontline HCWs to recover between waves, more so amongst those working directly in COVID-19 units. This has led to significant psychological issues as a consequence of the indirect impact of the disease. Extensive work has been published on the psychosomatic effects of COVID-19 on HCWs [6,7,8,9,10,11,12,13]. A systemic review with five studies that included 3257 HCWs reported stress, depression, anxiety and insomnia [6]. More interestingly, a meta-analysis and systemic review evaluated the outcomes of 13 studies with 33,062 HCWs showed 34% with insomnia in five studies, 23% with depression in 10 studies and a pooled prevalence of 23% anxiety in two studies [7]. A multicenter multinational study also reported that at least one symptom, psychological or physical was experienced by 67% of HCWs [8]. Amongst all negative mental health outcomes, one that holds the biggest red flag is “fear”; in fact, one study from Henan reported ‘fear of self-infection’ in up to as high as 85% of 1357 HCWs [12] and another from Egypt had similar findings of 83% [13]. Furthermore, this can be so debilitating that it previously led to refusal of working amongst HCWs during the human pandemic influenza without optimal conditions or immediate provisions of immunization and antiretroviral therapy, thus reducing the medical workforce by more than 50% [14]. Terms such as fear (self-infection or other uncertainties), stress or anxiety can be almost used interchangeably and at a psychological level maybe difficult to differentiate, since most HCWs may not freely admit to the term ‘fear’. Nevertheless this factor, undoubtedly leads to further regression in a medical personnel’s health and increased negative health outcomes. Our study done in the Czech Republic during the first phase of COVID-19 also evaluated the indirect effects of COVID-19 amongst otorhinolaryngologists in hospital-based as well as private practices and showed mental fatigue, burnout syndrome and physical tiredness. Other self-reported issues were fear, anger, uncertainty and confusion with the situation, dissatisfaction with the health care system, disrupted personal life and lack of insight to the future [11]. In addition, lack of support from employers was also another major issue found in our previous study [11]. We therefore decided to evaluate the current situation amongst doctors working in dedicated COVID-19 units during the second and stronger phase of COVID-19 in the Czech Republic; we also wanted to ascertain the debilitating issues and if necessary, offer possible solutions towards improvement of the situation.

## 2. Materials and Methods

### 2.1. Study Aim, Questionnaire Design and Distribution

A prospective questionnaire-based study was created using google questionnaire with 14 mandatory questions to address relevant matters for doctors working in the COVID-19 units. The first four questions were based on demographic information gender (male; female); specialty where primarily employed (18 options were available); years of work experience after completion of medical school (0–5; 6–10; 11–20; 21–35; >35) and lastly if the doctor volunteered or was transferred to a COVID-19 unit. Following which, the questionnaire was divided into two areas of interest, the first part of the survey mainly dealt with the adequacy of provisions at the COVID-19 as well as work related issues and the second studied the psychosomatic status of the doctors in response to COVID-19, support provided by employers along with strategies in reducing COVID-19 in the Czech Republic. Responses were in the form of a drop-down menu and the single best answer was selected for questions 1–13, whilst question no. 14 had the option to select as many responses as possible. Only one question had the additional possibility to comment. For the purposes of this article, the second part of the survey (Table 1) was analyzed and discussed.

We approached the Czech Medical Association through the Czech Society of otorhinolaryngology and head and neck surgery to obtain permission and help us distribute the questionnaire to the chairpersons of 18 medical and surgical Czech societies and in turn to the members of their respective societies. An invitation email with an online link to the questionnaire was sent to all the societies. Every society forwarded the email to the doctors in their respective specialties. A time period of 31 days (15 January 2021–18 February 2021) was allocated for completing the questionnaire.

### 2.2. Participants Selection Criteria

Inclusion criteria: (1) doctors working or worked as frontline workers in a COVID-19 unit; (2) all invited medical and surgical societies; exclusion criteria: (1) any specialist society that refused or did not respond to the invitation.

### 2.3. Data Analysis

Responses from the questionnaire was collected as data on Microsoft Excel. Simple descriptive statistical analysis was carried out. Comparison of demographic data to psychological impact of working in a COVID-19 unit and resulting psychosomatic problems was done to establish a cause and effect relationship. Furthermore, frequency of positivity of SARS-CoV-2 antigen and course of COVID-19 disease amongst doctors working in COVID-19 units was analyzed to interpret the risk. Lastly, evaluation of current support provided by employer if doctors contract COVID-19 versus type of support expected by doctors as well as suggested strategies for reducing COVID-19 in the Czech Republic were discussed here.

### 2.4. Compliance with Ethical Standards

Institutional Review Board Statement: Formal ethical approval was not required for this survey since it was questionnaire-based. Protocol followed in studies involving human subjects were in compliance with the Helsinki declaration and further in accordance with local ethical guidelines of the institutional ethical committee Charles University, Prague, the Czech Republic. Furthermore, it was also approved by the Czech Medical Association.

## 3. Results

Fifteen specialties agreed to participate. These specialties were divided into medical and surgical units. Anesthesiology, resuscitation and intensive care medicine, gerontology and geriatrics, infectious diseases, internal medicine, neurology, oncology, pediatrics, pneumonology and psychiatry were categorized as medical specialties whilst cardiovascular surgery, general surgery, neurosurgery, orthopedics and traumatology, otorhinolaryngology and head and neck surgery as well as urology were characterized as surgical units. A total of 225 doctors (125 males; 100 females) sent completed responses. One hundred and seven (50 males; 57 females) doctors were from medical specialties, whereas 118 (75 males; 43 females) doctors were from surgical specialties.

The psychological impact of working in a COVID-19 unit varied by gender and specialty. The highest frequency on moderate impact was seen amongst male doctors from surgical units, whilst extreme impact was perceived more by females from medical units (Table 2). With regard to the most serious impact of COVID-19 on doctors working in the COVID-19 units, most were concerned with health (self or relative) issues.

Most doctors experienced moderate to severe degrees of psychological impact of working in a COVID-19 unit.

**Figure 1:**
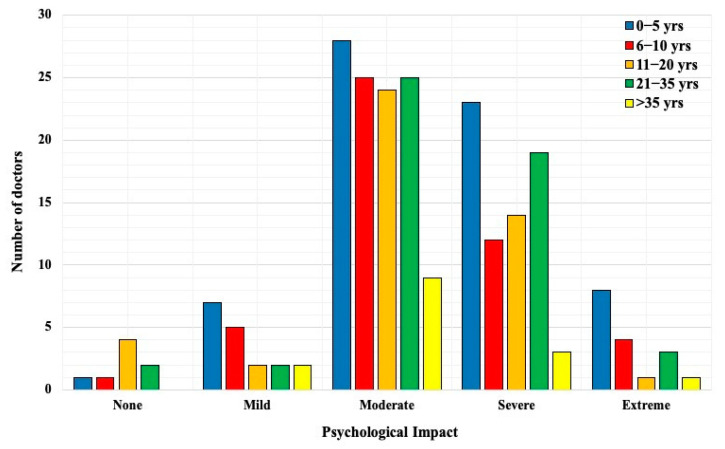
Comparison of work experience and psychological impact of working in a COVID-19 unit.

Highest impact was seen amongst those with 0 to 5 years of experience and secondly amongst those with 21 to 35 years of experience (Figure 1).

**Figure 2:**
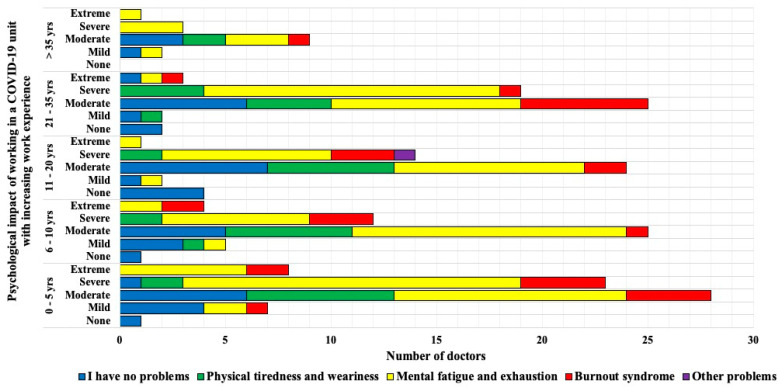
Comparison of psychological impact of working in a COVID-19 unit with increasing work experience and psychosomatic effect.

In general, those with moderate to severe psychological problems with working in a COVID-19 unit experienced more psychosomatic issues (Figure 2). Mental fatigue was noted in most who perceived extreme psychological impact of working in a COVID-19 unit. The most vulnerable age groups were those with least working experience and those with at least 21 to 35 years of experience.

A total of 89 doctors tested positive for the SARS-CoV-2 antigen test, amongst these 17 were asymptomatic and quarantined, 51 doctors suffered mild symptoms, 21 had moderate symptoms and none suffered severe problems. None of the doctors were hospitalized. Amongst the 136 doctors with negative test, twenty-three were underwent quarantine.

If a doctor contracted COVID-19, at least 114 doctors (52 medical, 62 surgical) stated that they had no support from their employers and about 58 doctors were unaware of any services. Otherwise 23 doctors selected that health services were available, 16 doctors had financial support and 14 had both health and financial employers’ support. More than 50% of all doctors suggested that all (psychological counselling, medical and financial) support should be provided (Table 3).

In the last question, doctors were asked to select as many options as they wanted for suggested approaches in controlling the situation with COVID-19 in the Czech Republic (Table 4).

## 4. Discussion

Amongst 225 doctors, a total of 96 from surgical units and 79 doctors from medical specialties were transferred to COVID-19 units, whilst 50 volunteered from both fields.

This survey was carried out during a very critical period and during the second and stronger wave, when the Czech Republic faced highest number of COVID-19 cases.

With regard to the most serious impact of COVID-19 on doctors working in the COVID-19 units, approximately 35% of all doctors had health (self and family) concerns. This is comparable to the survey we did during the first phase of COVID-19 in the Czech Republic, where 38.7% doctors had the same concern. Furthermore both our surveys report similar patterns of moderate psychological impact amongst 50% of all doctors [11]. This shows that COVID-19 has a strong psychological impact on doctors even when a lot about the disease has been discovered since March 2020, when it was first detected in this country. Prevalence of ‘fear of self-infection’ has been reported as high as above 80% amongst HCWs in certain studies [12,13]. The study conducted amongst frontline workers in Egypt also showed that 89.2% HCWs stated that they were more susceptible to COVID-19 infection in comparison to others [13]. In this study, the two work experience groups that were greatly influenced by this disease were the youngest with 0 to 5 years and those with 21 to 35 years of experience. This in turn, also made an impact on psychosomatic problems faced during work. Mental fatigue was the most commonly noted problem and was highly perceived by those suffering extreme psychological impact of working in a COVID-19 unit. Therefore, this may be regarded as a directly proportional relationship. Burnout syndrome was seen frequently amongst the youngest group. Studies showed similar findings that frontline workers and young age are more susceptible to severe mental health problems including anxiety, depression, post-traumatic stress disorder, and sleep disorders [6,7,8,9,10,11]. On the contrary and interestingly, one study from Germany reported that their health professionals are less psychologically burdened than non-health professionals and also less burdened compared with existing international data [15]. A very important part of support in such cases is psychological counselling. This has been offered in certain hospitals, but was not mandatory and HCWs were not properly made aware of it. The mental exhaustion that surrounds one’s family or working environment (fear of infection, working in an alien field of medicine with inadequate training, wearing personal protective equipment for long hours, unsafe working environment, constant change in methodology and protocols, death of a patient or colleague, and so on) are numerous therefore mandatory counselling should be instituted to prevent further decrement in the wellbeing of a doctor or HCW. Other factors that can further worsen psychological condition are job insecurity, long periods of isolation, and uncertainty of the future; it is especially seen in younger people and in those with a higher educational background [10].

Around 40% of doctors in our survey tested positive for the COVID-19 virus. This is an alarming finding even in a small cohort. However, our results are in concurrence with a systemic review and meta-analysis conducted on peer-reviewed articles between 1 January and 9 July 2020, that looked at SARS-CoV-2 only amongst HCWs. This study analyzed 58 out of 328 articles found, a very detailed analysis of clinical manifestations, complications and outcome were discussed amongst 119,338 HCWs. It concluded that 51.7% of HCWs tested positive for COVID-19 with prevalence of hospitalization amongst 15.1% and mortality in up to 1.5% of HCWs [16]. In our study, none of the doctors were hospitalized. Most doctors have reported that no support was given by employers whilst working in COVID-19 units and expect all services to be provided. The Ministry of Health in the Czech Republic paid out two single payments as compensation to all HCWs irrespective of frontline or second-line or others for working corresponding to the two waves of COVID-19. No contracts or conditions were specifically made out for those working in COVID-19 units or those in high risk specialties. Therefore, this is not considered as ample compensation by most doctors. Health facilities offered to employees contracting the disease were similar to those with the public and according to standard protocols of each hospital. In terms of sick leave, one study that studied legal systems for managing workplace COVID-19 infection risk in Asia-Pacific countries reported that although these countries maintain legal systems that govern the duration, administration and financing of paid sick leave, many workers may not have access to it even if legally guaranteed [17]. We should further consider if the disease is contracted due to an occupational hazard or as an occupational illness, then employees might expect further damage compensation since there is no standardized increment in salary. The major flaw is the lack of specific clauses or articles defining mitigating virus risk in a workplace that are required by employers, making it also difficult for employers to act on such circumstances [17]. This also brings up another major legal issue on death of an employee related to occupationally acquired COVID-19; although all employers have obligations to pay under such circumstances, source of infection and an appeal for such compensation may not be an easy procedure. These uncertain circumstances further contribute to the indirect health consequences of the disease. During such difficult times, HCWs from other countries might be facing similar problems, however alterations should be made since HCWs especially doctors are working completely outside their fields of practice and risking their careers and lives on a daily basis.

Lastly, the Czech Republic registered the highest per capita rate of new COVID-19 cases in the world during January 2021, therefore it was the most appropriate time to evaluate strategies in improving the COVID-19 situation in the Czech Republic. A total of 196 doctors selected ‘Preventive measures in combination with vaccination’, 82 selected ‘Private doctors should, if necessary, supplement hospital staff in order to maintain the efficient operation of hospitals in a pandemic situation’, 74 chose ‘Nonadherence to lockdown rules and preventive measures resulting in imprisonment or penalty’, 61 thought ‘Dividing the Czech Republic into different categories of risk zones and enforcing lockdown and other measures accordingly’, 60 opted for ‘Stricter guidelines to general practitioners on referral of patients to hospitals’, 60 for ‘utilization of telemedicine’ and 13 thought ‘All of the above’ would be helpful. Our previous survey also helped formulate recommendations made for public, patients, medical staff and employers [18]. The study that compared legal measures and governance established for managing COVID-19 infection in Asia-pacific countries (Indonesia, India, Japan, Malaysia, New Zealand, Republic of Philippines, Republic of Korea, Taiwan and Thailand), also made legal recommendations. These include the provision of adequate personal protective equipment for all employees; proper environmental measures (engineering control measures by type of work environment, including ventilation, partitions, booths and as recommended by individual Industrial Safety and Health Acts); administrative measures that reinforce education, social distancing and individual hygiene measures; and lastly legal and social protection for HCWs who contract COVID-19 infection [17].

The COVID-19 disease is currently in the 2nd wave in most countries and in quite a few, a 3rd wave has also started. History shows us that a pandemic related to a single infectious agent can have multiple waves and reappear centuries later, as seen with the ‘black death’ or plague. This led to 18 waves in the first pandemic, wiped off one-third of Europe in the 2nd pandemic and killed off an estimated 200 million people through further attacks [19,20]. An article from USA reported reduction in plague related fatalities from 66% to 13% with the discovery of antibiotics in 1942 [21], thus controlling the disease spread. In current times, with the advancement of medical sciences, the debacle that occurred centuries ago has thankfully not occurred with COVID-19. However, the direct and indirect damages related to the disease are also irreparable and of enormous magnitude. This historical disease has resurfaced so many centuries later, with the most recent cases of plague reported from California in 2015 [22] and Mongolia in 2020 [23]. This implies that there is no guarantee that these infectious agents may not also be used for the purposes of bioterrorism, therefore all we can hope for is controlling or isolating an infectious agent to prevent spread or misuse.

We conducted our study amongst doctors working in COVID-19 units in a very critical period during the second and much stronger phase of COVID-19, hence responses might have been slightly lower than expected. Although the main psychosomatic effects were addressed here and there was an option to comment, due to the compact study design and no comments received, other possible psychosomatic issues seen and reported in previous studies were not fully explored.

However, the aims of formulating recommendations and suggesting strategies are not only for preventing the risk of developing new and more virulent strains of the SARS-CoV-2 virus, future waves, but delaying it and/or reducing the ferocity thus controlling the morbidity and mortality associated with the disease. It is also targeted at adequately protecting and supporting HCWs in such situations. Thus in turn, will ease the workload and reduce debilitating health effects faced currently by COVID-19 workers, especially psychosomatic effects.

## 5. Conclusions

In the Czech Republic, a country with 10.7 million population, around 1.67 million cases and 30,298 COVID-19 related deaths have been recorded till date.

This study reiterates the importance of appropriate training of HCWs, institution of mandatory and legal guidelines for employers as well as employees, easy access to relevant services and information, adequate support and compensation for HCWs, as well as practical application of strategies for prevention and control of disease. During previous pandemics and historical times, like one seen in the 14th century, unfortunately, due to the virulence of the disease and limited medical services, nearly all of the so-called ‘Plague doctors’ perished from the infectious consequences of the disease. Although we prevented this largescale tragedy amongst ‘COVID-19 doctors’ due to advancement in healthcare, the indirect psychosomatic affliction from the disease have been overwhelming. Subsequent waves, inadequate support as well as unparalleled workload can lead to a serious rise in both incidence and prevalence of psychological disorders amongst HCWs worldwide. We recommend a follow-up study to evaluate the situation after implementation of suggested changes.

## Figures and Tables

**Table 1:** Sample questionnaire outlining second part of survey.

Type of Question	Options for Responses
How do you perceive the psychological impact of working in a COVID-19 unit?	5—point Likert scale from None to Extreme
Do you have any psychosomatic problems whilst working in the COVID-19 unit?	5 options were available (select 1 option) □ No problems □ Physical tiredness and weariness □ Mental fatigue and exhaustion □ Burnout syndrome □ Other problems * If selected other problems, option to comment was available
Have you tested positive for SARS-CoV-2 antigen or were you quarantined whilst working in the COVID-19 unit?	□ Yes □ No * If selected ‘yes’, appropriate course of disease to be selected from 4 options (□ quarantined/not □ mild □ moderate □ severe) * If selected ‘no’, select if quarantined or not
What support is provided by employer If an employee contracts COVID-19 disease?	5 options were available (select 1 option) □ unaware □ none □ medical □ financial □ medical + financial
What services should be provided by employers for employees working at a COVID-19 unit?	5 options were available (select single best option) □ unaware □ psychological counselling □ medical □ financial □ all of the above
In your opinion, what strategies could help improve the situation with COVID-19 in Czech Republic?	9 options were available (select as many as applicable)

**Table 2:** Psychological impact of working in a COVID-19 unit.

Specialty	Gender	Severity				
		None	Mild	Moderate	Severe	Extreme
Medical	Males	2	6	27	13	2
	Females	2	2	22	18	13
Surgical	Males	4	8	40	22	1
	Females	0	2	22	18	1

**Table 3:** Suggested services to be provided for employees at COVID-19 units.

Specialty	Type of Support				
	Unaware	Psychological Counselling	Medical	Financial	All Services
Surgical	6	8	6	14	84
Medical	6	13	0	9	79

**Table 4:** Strategies that can help improve the situation with COVID-19 in the Czech Republic.

Option	Strategies in Reducing COVID-19 in Czech Republic	Respondents
1	Preventive measures in combination with vaccination	196
2	Private doctors should, if necessary, supplement hospital staff in order to maintain the efficient operation of hospitals in a pandemic situation	80
3	Nonadherence to lockdown rules & preventive measures resulting in imprisonment or penalty	74
4	Dividing Czech Republic into different categories of risk zones and enforcing lockdown and other measures accordingly	61
5	Stricter guidelines to general practitioners on referral of patients to hospitals	60
6	Utilizing telemedicine	60
7	Reinforcing travel restrictions and quarantine guidelines	20
8	All of the above	13
9	None of the above	3

## Data Availability

Available on request.
